# Increased Ascorbate Content of Glioblastoma Is Associated With a Suppressed Hypoxic Response and Improved Patient Survival

**DOI:** 10.3389/fonc.2022.829524

**Published:** 2022-03-28

**Authors:** Eleanor R. Burgess, Rebekah L. I. Crake, Elisabeth Phillips, Helen R. Morrin, Janice A. Royds, Tania L. Slatter, George A. R. Wiggins, Margreet C. M. Vissers, Bridget A. Robinson, Gabi U. Dachs

**Affiliations:** ^1^ Mackenzie Cancer Research Group, Department of Pathology and Biomedical Science, University of Otago Christchurch, Christchurch, New Zealand; ^2^ Metastasis Research Laboratory, GIGA-Cancer, University of Liège, Liege, Belgium; ^3^ Cancer Society Tissue Bank, University of Otago Christchurch, Christchurch, New Zealand; ^4^ Department of Pathology, Dunedin School of Medicine, University of Otago, Dunedin, New Zealand; ^5^ Centre for Free Radical Research, Department of Pathology and Biomedical Science, University of Otago Christchurch, Christchurch, New Zealand; ^6^ Canterbury Regional Cancer and Haematology Service, Canterbury District Health Board, and Department of Medicine, University of Otago Christchurch, Christchurch, New Zealand

**Keywords:** glioma, HIF-1, VEGF, vitamin C, glioblastoma multiforme, 2-oxoglutarate dependent dioxygenases, HIF-hydroxylase

## Abstract

Glioblastoma multiforme is a challenging disease with limited treatment options and poor survival. Glioblastoma tumours are characterised by hypoxia that activates the hypoxia inducible factor (HIF) pathway and controls a myriad of genes that drive cancer progression. HIF transcription factors are regulated at the post-translation level *via* HIF-hydroxylases. These hydroxylases require oxygen and 2-oxoglutarate as substrates, and ferrous iron and ascorbate as cofactors. In this retrospective observational study, we aimed to determine whether ascorbate played a role in the hypoxic response of glioblastoma, and whether this affected patient outcome. We measured the ascorbate content and members of the HIF-pathway of clinical glioblastoma samples, and assessed their association with clinicopathological features and patient survival. In 37 samples (37 patients), median ascorbate content was 7.6 μg ascorbate/100 mg tissue, range 0.8 – 20.4 μg ascorbate/100 mg tissue. In tumours with above median ascorbate content, HIF-pathway activity as a whole was significantly suppressed (p = 0.005), and several members of the pathway showed decreased expression (carbonic anhydrase-9 and glucose transporter-1, both p < 0.01). Patients with either lower tumour HIF-pathway activity or higher tumour ascorbate content survived significantly longer than patients with higher HIF-pathway or lower ascorbate levels (p = 0.011, p = 0.043, respectively). Median survival for the low HIF-pathway score group was 362 days compared to 203 days for the high HIF-pathway score group, and median survival for the above median ascorbate group was 390 days, compared to the below median ascorbate group with 219 days. The apparent survival advantage associated with higher tumour ascorbate was more prominent for the first 8 months following surgery. These associations are promising, suggesting an important role for ascorbate-regulated HIF-pathway activity in glioblastoma that may impact on patient survival.

## Introduction

Glioblastomas (World Health Organisation (WHO) grade IV) are an incurable brain cancer with survival measured in months not years, despite modern, multimodal treatment (1–4). Median age at diagnosis for glioblastoma is 59-64 years, with only a 5.5% 5-year survival rate, compared with lower grade (WHO II-III) gliomas that are diagnosed at a younger age (age 36-53 years) and have a 5-year survival rate of 30-83% (4, 5). Standard treatment for glioblastoma consists of maximal debulking surgery followed by chemotherapy and/or radiotherapy, with the aim of relieving neurological symptoms and maintaining patient quality of life (6–8). Poor outcome and limited treatment options for patients with glioblastoma highlight the urgent need for additional therapeutic strategies.

Glioblastomas are an aggressive type of glioma (tumours that arise from glial cells) that make up over half of all malignant adult primary brain tumours (5, 9). Gliomas also encompass the lower-grade astrocytomas and oligodendrogliomas. Classification of gliomas is moving from histopathological criteria to molecular profiling based on genomics, transcriptomics and epigenomics (4, 10–12). Although the current WHO 2016 classification still uses the histopathological grading system, molecular markers (isocitrate dehydrogenase (IDH1/2) and chromosome 1p and 19q codeletion) are now frequently utilised for diagnosis and prognosis (11). IDH status is prognostic for patient outcome, with the presence of IDH mutations being associated with a more favourable prognosis than IDH wild-type tumours; patients with IDH wild-type tumours have a median overall survival of 14 months compared to IDH mutant with 43 months (4). In addition, IDH mutant glioblastomas have lost the transcriptional regulator, ATP-dependent helicase, which is retained in IDH wild-type glioblastoma (10). The European Association of Neuro-Oncology guidelines have recently proposed discontinuation of the term ‘IDH mutant glioblastoma’, instead calling these ‘astrocytoma grade 4’ (10). However, in this study we will use the term glioblastoma (WHO grade IV) to describe both IDH wild-type and IDH mutant tumours.

Ascorbate has been suggested as a complementary treatment option for glioblastoma, but there is no robust clinical evidence to support this notion (13–15). Ascorbate acts as a co-factor for the 2-oxoglutarate dependent dioxygenase (2-OGDD) enzymes (16, 17). The 2-OGDD superfamily of enzymes also requires 2-oxoglutarate (2-OG or α-ketoglutarate, generated *via* IDH activity (IDH1 in the cytoplasm, IDH2 in the mitochondria (4)) in the Krebs cycle) and oxygen as substrates, and ferrous iron as an additional co-factor for optimal function (16). A reduction in any of the substrates or co-factors leads to reduced enzyme function (17–21).

Reduced oxygen levels, a key characteristic of glioblastoma tumours (22–24), lead to activation of the hypoxic response, driven by the hypoxia inducible factors 1-3 (HIF-1-3). HIFs are heterodimer transcription factors, consisting of a constitutively expressed β-subunit and regulatory α-subunits (HIF-1α, HIF-2α, HIF-3α) (25–27). Under physiological oxygen levels, HIF-α is hydroxylated by HIF-hydroxylases, prolyl hydroxylase (PHD1-3) and factor inhibiting HIF (FIH), all members of the 2-OGDD superfamily (18, 19, 28, 29). PHDs hydroxylate two proline residues on HIF-α, targeting it for proteasomal degradation, whereas FIH hydroxylates an asparagine residue, preventing coactivator binding and reducing transcriptional activity (29, 30). Hypoxia leads to reduced HIF-hydroxylase activity, resulting in accumulation of the HIF-α subunit and activity, and subsequent formation of the HIF transcription factor (16, 31, 32). The HIF transcription factors target genes containing hypoxic response elements (HRE), inducing gene expression (25, 33). HIF target genes are involved in tumour formation, progression, invasion, metastasis and treatment resistance (33–35). In glioblastoma, HIF expression and activity have been linked to a more aggressive cancer phenotype and poor survival (36–42).

The ability of ascorbate to reduce hypoxic pathway activity has been verified in numerous *in vitro* (43–45) and *in vivo* studies (46–48). An association between higher ascorbate and lower HIF activity has also been demonstrated in human clinical tumour samples from endometrial, colorectal, kidney, breast and thyroid cancer (49–53). Although these associations are persuasive, they do not establish a causative relationship. The first tentative evidence was recently provided in a small intervention trial of patients with colorectal cancer, that demonstrated that high dose ascorbate infusions were able to penetrate tumour tissue and reduce hypoxic pathway activity (54).

Levels of ascorbate in brain tissue are among the highest in the human body (55–57). However, data on ascorbate in human brain cancer is sparse. To our knowledge, only one previous study has measured ascorbate in low-grade glioma (n=11), reporting that astrocytoma tissue contained lower ascorbate levels than matched normal brain tissue (58). Thus, data on ascorbate concentrations in glioblastoma is lacking, and the relationship between ascorbate and the hypoxic pathway has not been explored in brain cancer.

The aim of this study was to investigate the hypoxic pathway and its relationship to tumour ascorbate levels in glioblastoma. Here we present data from 37 human glioblastoma tissue samples analysed for ascorbate content and protein levels of HIF-1α and selected down-stream targets, along with patient clinical information and follow-up data.

## Materials and Methods

### Materials

All chemicals were from Sigma-Aldrich (St Louis, MO, USA), unless stated otherwise.

### Ethics and Patient Consent

Ethics approvals from the University of Otago Ethics Committee (H19/163) and the national Health and Disability Ethics Committee (MEC/08/02/016) were obtained for this study. Approval for the use of samples was also obtained from the Canterbury Tissue Bank Board (2001DPVR). Informed written consent was given by each donor for the use of their sample and access to clinical records for research.

### Clinical Samples

Glioblastoma tumour samples were donated to the Cancer Society Tissue Bank (CSTB) and the University of Otago (Dunedin, New Zealand) following resection or debulking surgery. Surgeries were carried out at Christchurch Hospital, the main tertiary hospital on the South Island of New Zealand, and at Dunedin hospital, between 2003-2019. In total, 37 human glioblastoma samples from 37 patients (CSTB n = 26, University of Otago Dunedin n = 11 samples) were included in this study. Tissue collection and storage follows strict protocols to ensure sample integrity. Briefly, samples are snap frozen in liquid nitrogen within 40 min of surgery and stored at -80°C until use. This process was previously shown to preserve ascorbate and proteins for analysis (49). Clinical notes provided information on patient demographics and clinicopathological data. Tumour size was estimated from imaging (computerized tomography and/or magnetic resonance imaging). Necrosis and vascular proliferation data were obtained from histology reports, and tumour location from surgical notes.

### Patient Treatment and Follow-up

Patients with glioblastoma were managed at Christchurch or Dunedin hospitals according to nationally accepted guidelines. Tumours were resected (debulked), followed by fractionated radiotherapy (60 Gy) or chemoradiation (60 Gy with temozolomide). The patient cohort was unselected, and there was no intervention or information regarding dietary/supplement for this cohort. Patient follow-up data and treatment was collated from clinical notes. Disease-free survival was calculated with the date of reported recurrence by imaging as endpoint. Mortality was calculated from the date of primary surgery to the date of death, presumed from brain cancer.

### Tissue Processing

Processing and extraction procedures were optimized using mouse brain tissue (Tissue Retrieval form G, University of Otago Animal Ethics Committee). Approximately 30 mg of frozen tissue sample was ground to a fine powder in liquid nitrogen using a pre-chilled mortar and pestle on dry ice. Powder was divided into three parts and weighed. One part was used for ascorbate analysis, one for DNA content and ELISA, and one for Western blotting; each containing approximately 10 mg of tissue.

### Ascorbate Analysis

Tissue powder was homogenised with a ground glass pestle in ice cold potassium phosphate buffer (pH 7.4) and immediately mixed with an equal volume of 0.54 M perchloric acid containing ~100 µM diethylenetriaminepentaacetic acid and incubated on ice for 10 minutes. Protein was pelleted by centrifugation at 12,000 g for 2 minutes and the supernatant containing ascorbate was stored at -80°C. Immediately prior to ascorbate analysis, samples were reduced using tris(2-carboxyethyl)phosphine (32 mM final concentration) for 3 hours at 4°C. Ascorbate was measured using reverse phase high performance liquid chromatography with electrochemical detection (HPLC-ECD), as described previously (49, 52, 59). A freshly made ascorbate standard curve (1.25-40 µM sodium-L-ascorbate) was run with each HPLC cycle and used to ascertain ascorbate concentrations. Ascorbate data was presented as tissue ascorbate (normalised to tissue weight) and cellular ascorbate (normalised to sample DNA content).

### DNA Content

Genomic DNA was purified from frozen glioma powder using the PureLink™ Genomic DNA Kit (Thermo Fisher Scientific, Auckland, NZ) following the manufacturer’s instructions for extraction from mammalian tissues. Total concentration of extracted genomic DNA was determined using Qubit™ dsDNA HS Assay (Thermo Fisher Scientific).

### IDH1 Mutation Status

Exon 4 of *IDH1*, which contains the R132 codon, was amplified from purified tumour DNA using target-specific primers (IDH1 Fwd: 5′-AATGAGCTCTATATGCCATCACTG, IDH1 Rev: 5′-TTCATACCTTGCTTAATGGGTGT). The PCR conditions were: 95°C for 2 min, followed by 35 cycles of 94°C for 30 s, 58°C for 30 s, 72°C for 30 s, and a final extension at 72°C for 10 min with TAQ-Ti DNA Polymerase (Fisher Biotec. Wembley, WA, Australia). An internal sequencing primer (IDH1 Seq: 5′-CCATTATCTGCAAAAATATC) was used to Sanger sequence (Genetic Analysis Service, University of Otago) each amplicon and the R132 codon was genotyped by manual inspection of corresponding chromatograms.

### Western Blotting

Tissue powder was homogenised in RIPA buffer (50 mM Tris (pH 8), 150 mM NaCl, 1% IGEPAL, 0.5% sodium deoxycholate, 0.5% sodium dodecyl-sulphate (SDS)) containing Complete Protease Inhibitor Cocktail (Roche, Auckland, NZ). Homogenates were mixed with sample buffer (60 mM Tris pH 4.8, 2% SDS, 20% glycerol, 0.01% bromophenol blue, 0.1 M dithiothreitol) and loading was standardised to 0.4 µg DNA per lane. Proteins were separated on 4-12% gradient Bis-Tris Plus SDS gels using 125 V for 75 minutes. Proteins were transferred to 0.45 µm polyvinylidene difluoride membranes using 25 V for 60 minutes. A cell lysate positive control (MDA-MB-231 cell line treated with 50 μM cobalt chloride, a hypoxia mimetic, for 4 hours) was run on all gels to allow for interblot standardisation. Membranes were blocked using 5% skim milk or 3% BSA and incubated overnight at 4°C with primary antibodies diluted in blocking solution. Antibodies were against hypoxia inducible factor 1 (HIF-1α, 1:800, AF1935, R&D Systems, Minneapolis, MN, USA), hexokinase 2 (HKII, 1:1000, Ab209847, Abcam, Cambridge, UK), carbonic anhydrase-9 (CA-IX, 1:200, R&D Systems AF2188), BCL2/adenovirus E1B 19 kDa protein-interacting protein 3 (BNIP3, 1:1000, R&D Systems AF4147), phosphoglycerate kinase 1 (PGK1, 1:5000, Abcam Ab38007) and glucose transporter 1 (GLUT1, 1:1000, Abcam Ab14683). Mouse brain samples were analysed using anti-N-cadherin antibody (1:1000, Abcam Ab76057), as hypoxia pathway proteins are low or undetectable in normal tissue. Membranes were incubated with horseradish peroxidase-conjugated secondary antibodies (anti-rabbit, anti-goat or anti-mouse) for 1 hour at room temperature and protein bands visualised using ECL Select Western Blotting Detection Reagent (RPN2235, Cytiva) and the NineAlliance imaging system (Uvitec). Glyceraldehyde 3-phosphate dehydrogenase (GAPDH, 1:5000, Abcam ab181602) and β-actin (1:5000, Sigma A5441) were assessed as loading controls, and total protein loading was determined using Coomassie blue staining of membranes. Band density was interpreted using the NineAlliance system. Protein standardisation was assessed using GAPDH and β-actin, with β-actin chosen as representative of total protein loading (**Supplementary Figure 1**).

### ELISA

Tissue powder was homogenised in ice cold 10 mM potassium phosphate (pH 7.4) and analysed for vascular endothelial growth factor A (VEGF) protein levels using the human VEGF Quantikine ELISA kit (SVE00, R&D Systems) following manufacturer’s instructions.

### Hypoxic Pathway Score

Relative protein levels for each hypoxic protein were attained by normalising band density to β-actin to correct for loading, and to the positive control to correct for differences in development between blots. To obtain a relative hypoxic pathway score for each tumour, the expression of each protein was divided into the top, middle and bottom third of the data (top 1/3 of cohort = 3, middle 1/3 of cohort = 2, lowest 1/3 of cohort = 1), protein categories for each patient were added together and divided by the number of proteins assessed, which provided a relative hypoxic pathway score for each patient.

### Statistical Analysis

Data were analysed using GraphPad Prism (9.2.0) with significance set at p<0.05. All data was analysed for normality using the Shapiro-Wilk test. Relationships between ascorbate, the HIF pathway and clinicopathological variables were assessed using unpaired Mann-Whitney or t-tests, for non-parametric and parametric data, respectively. Spearman’s and Pearson’s correlations were used to test associations between clinicopathological variables and ascorbate or the HIF-pathway score. Kaplan-Meier survival curves were analysed using the Gehan-Breslow-Wilcoxon test. Multiple groups were compared using Kruskal-Wallis, followed by Dunn’s multiple comparison test. Cox regression univariate and multivariate analyses were used to assess patient survival against ascorbate, the HIF-pathway score and other variables, using R (version 3.6.1).

## Results

### Patient and Tumour Characteristics

The patient cohort consisted of 37 patients, males made up 70% of the cohort, and the mean age at diagnosis was 60 years old, with 41% of patients being younger than 60. A majority of the cohort (89%) identified as NZ European (**Table 1**).

**Table 1:** Patient demographics.

Parameter	Number (%)
Total n	37 (100)
**Gender**	
Male	26 (70)
Female	11 (30)
**Age**	
<60 years	15 (41)
≥60 years	22 (59)
**Ethnicity**	
NZ/European	33 (89)
NZ/Maori	1 (3)
Other	3 (8)
**Treatment**	
Radiation only	12 (32)
Chemotherapy only	1 (3)
Chemoradiation	18 (49)
No treatment	4 (11)
Not recorded	2 (5)

Clinicopathological data for this cohort are shown in **Table 2**. All tumours were classified as glioblastoma (WHO grade IV), with one known to have progressed from lower grade glioma to glioblastoma. Most tumours were less than the mean of 70 mm in size (median size 45 mm), and almost all presented with necrosis and microvascular proliferation (**Table 2**). As these samples were largely collected in the early 2000’s, only limited molecular information was available, including IDH1/2, ATRX or 1p19q status. Due to its importance in prognosis, we have sequenced all tumours to assess IDH1 mutation status (IDH1^R132H^). Accordingly, the cohort consisted of three IDH1 mutant glioblastomas (astrocytoma grade 4) and 34 IDH wild-type glioblastomas (**Table 2**).

**Table 2:** Clinicopathological details of cohort according to ascorbate content.

Characteristic	Number (%)	Ascorbate <7.6 μg/100 mg	Ascorbate >7.6 μg/100 mg
**Patients**	**37 (100)**	**18**	**19**
Male	26 (70)	12	14
Age ≥60 years	22 (59)	13	9
**Histological type**			
Glioblastoma multiforme	37 (100)	18	19
**Presentation**			
Primary	26 (70)	13	13
Progression	1 (3)	0	1
Not recorded	10 (27)	5	5
**Grade**			
Grade IV (IDH1 wild-type)	33 (89)	17	16
Grade IV (IDH1 mutant)	3 (8)	0	3
Unknown	1 (3)	1	0
**Position**	[Left/right]		
Frontal	13 [7/6]	5	8
Parietal	8 [3/5]	7	1
Temporal	10 [7/3]	1	9
Occipital	2 [1/1]	2	0
Temporal/parietal, occipital/parietal	4 [3/1]	3	1
**Tumour size**			
<45 mm	12 (32)	6	6
≥45 mm	15 (41)	7	8
Not recorded	10 (27)	5	5
**Necrosis**			
Present	26 (70)	13	13
Absent	1 (3)	0	1
Not recorded	10 (27)	5	5
**Microvascular proliferation**			
Present	24 (65)	11	13
Absent	3 (8)	2	1
Not recorded	10 (27)	5	5

### Sample Integrity and Optimisation

**Figure 1:**
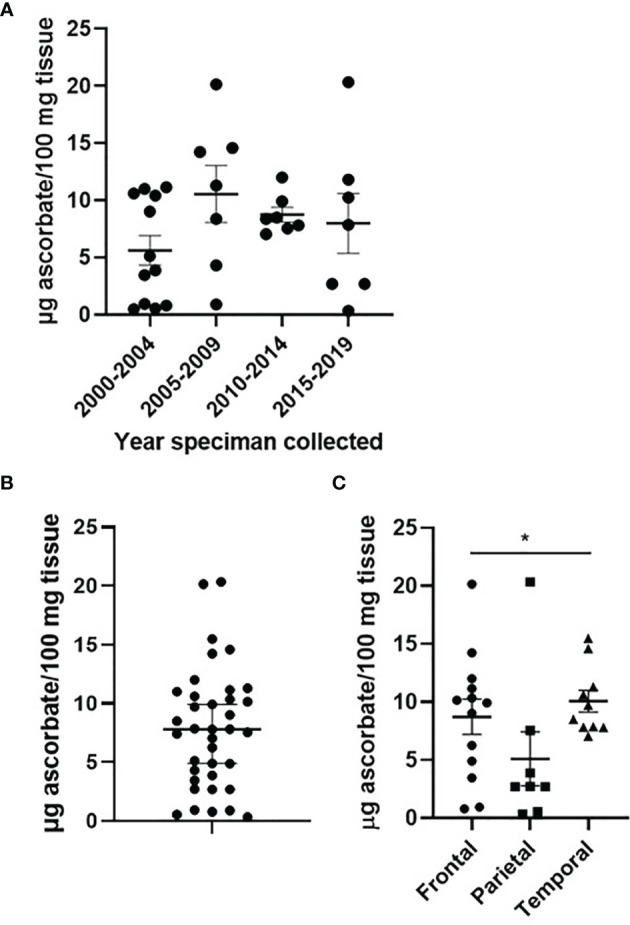
Ascorbate content of glioblastoma tumours. Human glioblastoma samples collected between 2000 and 2019, and processed in 2021, showed no significant change in ascorbate content **(A)**. Ascorbate was measured by HPLC-ECD and standardised to tissue weight **(B)**. Ascorbate content differed by tumour location within the brain **(C)**. n = 37 samples; mean ± SEM; *p < 0.05.

Human glioblastoma tissues had been stored at -80°C for 2-20 years. We investigated whether the tissue ascorbate content was affected by long-term storage: as shown in **Figure 1A**, there was no significant difference in the ascorbate content of human glioblastoma tissues over 20 years of storage. Optimisation experiments using mouse brain showed that ~10 mg of tissue was sufficient to obtain accurate ascorbate data, that processing of tissue in liquid nitrogen on dry ice preserved both ascorbate and protein integrity, and that samples frozen within 0-2 h from dissection showed no loss of ascorbate compared to samples analysed immediately (**Supplementary Figure 2**).

### Ascorbate Concentration of Clinical Glioblastoma Tissue

Ascorbate concentrations in glioblastoma tumours ranged from 0.8 – 20.4 μg ascorbate/100 mg tissue, with a mean of 7.61 and a median of 7.55 μg ascorbate/100 mg tissue (**Figure 1B**). When ascorbate was standardised to DNA content, the mean was 0.446 and the median 0.324 nmol ascorbate/μg DNA (**Supplementary Figure 3**).

Patient age (unpaired t-test, p = 0.058) and gender (p > 0.05) were not significantly different between tumours containing below or above median ascorbate (7.6 μg ascorbate/100 mg tissue) (**Table 2** **).** Tumour size (according to imaging), and necrosis and microvascular proliferation (according to histology) also did not differ by ascorbate content (**Table 2**). However, tumour location appeared to be distributed differently in the two ascorbate groups (below and above 7.6 μg ascorbate/100 mg tissue), with most above median ascorbate tumours located in the frontal or temporal lobes, whereas below median ascorbate tumours were more often located in the parietal lobes as well as spanning temporal/parietal and occipital/parietal boundaries (**Table 2**). There was a significant difference in ascorbate content of tumours located in the parietal vs frontal vs temporal lobes (mean 5.1, 8.7 and 10.1 μg ascorbate/100mg tissue, respectively, Kruskal-Wallis p = 0.041, **Figure 1C**). *Post-hoc* testing showed that both frontal and temporal lobe ascorbate levels were significantly higher than those of the parietal lobe tumours (Dunn’s multiple comparison test, p = 0.03 and 0.007, respectively).

### Hypoxic Pathway in Glioblastoma

**Figure 2:**
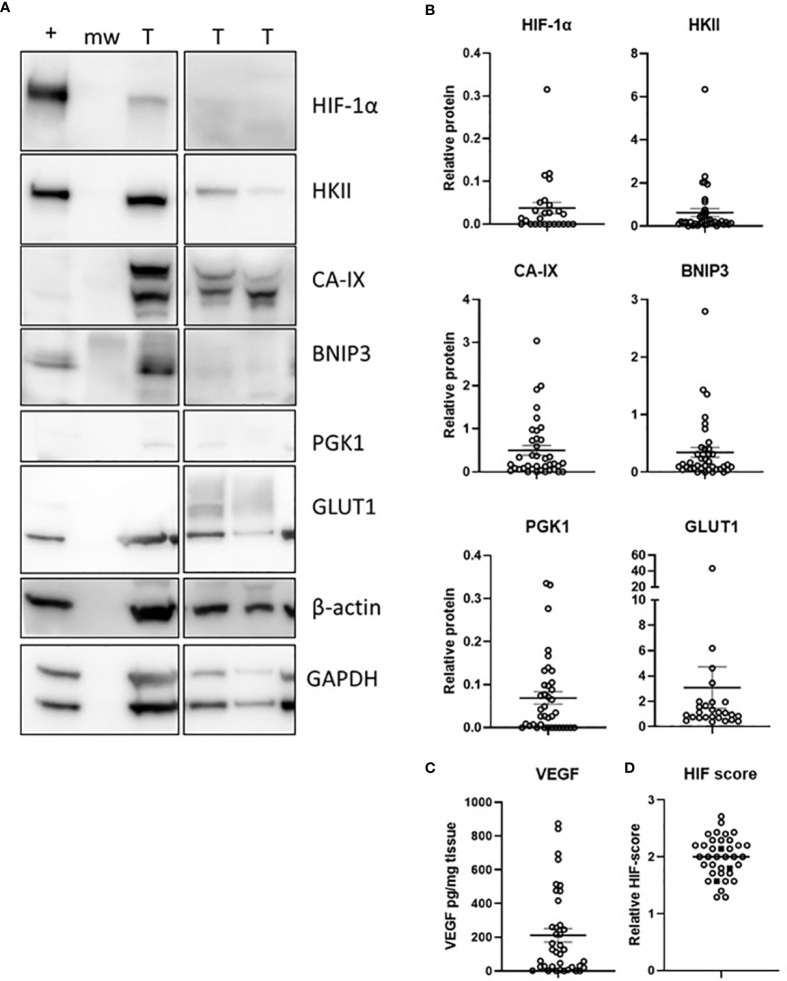
The hypoxic pathway in glioblastoma tumours. Levels of 7 HIF-pathway members were estimated by Western blotting **(A)**, with densitometry measures **(B)**, or measured by ELISA **(C)**. A HIF-pathway score was derived for each patient by combining the relative scores of 7 hypoxia-responsive proteins **(D)**; IDH1 mutant samples are shown as solid square symbols. n = 37 samples; T, tumour, +, positive control (MDA-MB-231 cell line exposed to 1% O_2_ for 16 h), mw, molecular weight marker; mean ± SEM.

Content of DNA (dsDNA) ranged from 0.056 – 0.742 μg DNA/mg tissue and was used to standardise sample loading for Western blots. Protein levels of HIF-1α and six selected downstream target proteins were measured using Western blotting (HIF-1α, HKII, CA-IX, BNIP3, PGK1, GLUT1, **Figure 2A**) or ELISA (VEGF). HIF-1α was detected in 15/37 samples, HKII in 36/37 samples, CA-IX (detected as a double band with the density of both bands combined for total protein) in 32/37 samples, BNIP3 in 34/37 samples, PGK1 in 27/37 samples and GLUT1 in 26/37 samples (**Figure 2B**). VEGF was detected in 33/37 samples and ranged from 0.65 – 874 pg/mg tissue (**Figure 2C**).

Associations between the different members of the HIF pathway were explored using Spearman’s correlation to determine pathway integrity in this disease (**Table 3**). BNIP3 was positively associated with HIF-1α (Spearman r 0.61, p = 0.0009) and HKII (Spearman r 0.43, p = 0.008), but negatively with PGK1 (Spearman r -0.38, p = 0.02). VEGF was positively associated with CA-IX (Spearman r 0.59, p = 0.0001) and PGK1 (Spearman r 0.34, p = 0.04). There were no significant relationships between any other hypoxic pathway proteins.

**Table 3:** Relationships between ascorbate and HIF-pathway proteins in glioblastoma.

		HIF-1α	HKII	CA-IX	BNIP3	PGK1	GLUT1	VEGF
**Tissue ascorbate^1^**	p	0.97	0.43	0.005 **	0.62	0.35	0.0039 **	0.26
	r	-0.007	0.14	-0.45	0.083	-0.16	-0.55	-0.19
**Cellular ascorbate^2^**	p	0.59	0.26	0.21	0.06	0.17	0.066	0.064
	r	0.11	0.19	-0.21	0.31	-0.23	**-**0.37	-0.31
**HIF-1α**	p		0.25	0.59	0.0009 ***	0.47	0.17	0.83
	r		0.23	-0.11	0.61	0.15	-0.28	-0.04
**HKII**	p			0.42	0.008 **	0.10	0.42	0.39
	r			0.14	0.43	-0.28	-0.17	-0.15
**CA-IX**	p				0.57	0.66	0.39	0.0001 ***
	r				0.10	0.08	0.18	0.59
**BNIP3**	p					0.02 *	0.19	0.17
	r					-0.38	-0.26	-0.23
**PGK1**	p						0.75	0.04 *
	r						0.07	0.34
**GLUT1**	p							0.37
	r							-0.18
**VEGF**	p							
	r							

^1^Tissue ascorbate, μg ascorbate/100 mg tissue.

^2^Cellular ascorbate, nmol ascorbate/μg DNA.

Bold shows significant p-values. *p < 0.05, **p < 0.01, ***p < 0.001.

The HIF pathway activity was assessed by combining the relative expression scores of the hypoxia-responsive proteins into one HIF pathway score for each patient (**Figure 2D**). There was no association between the clinicopathological variables (tumour size, necrosis, microvascular proliferation) and the HIF pathway (results not shown), likely because size was estimated by imaging and virtually all tumours were necrotic with microvascular proliferation. The three IDH1 mutant tumours had HIF pathway scores similar to IDH1 wild type samples (**Figure 2D**).

### Association Between Ascorbate and the HIF Pathway

**Figure 3:**
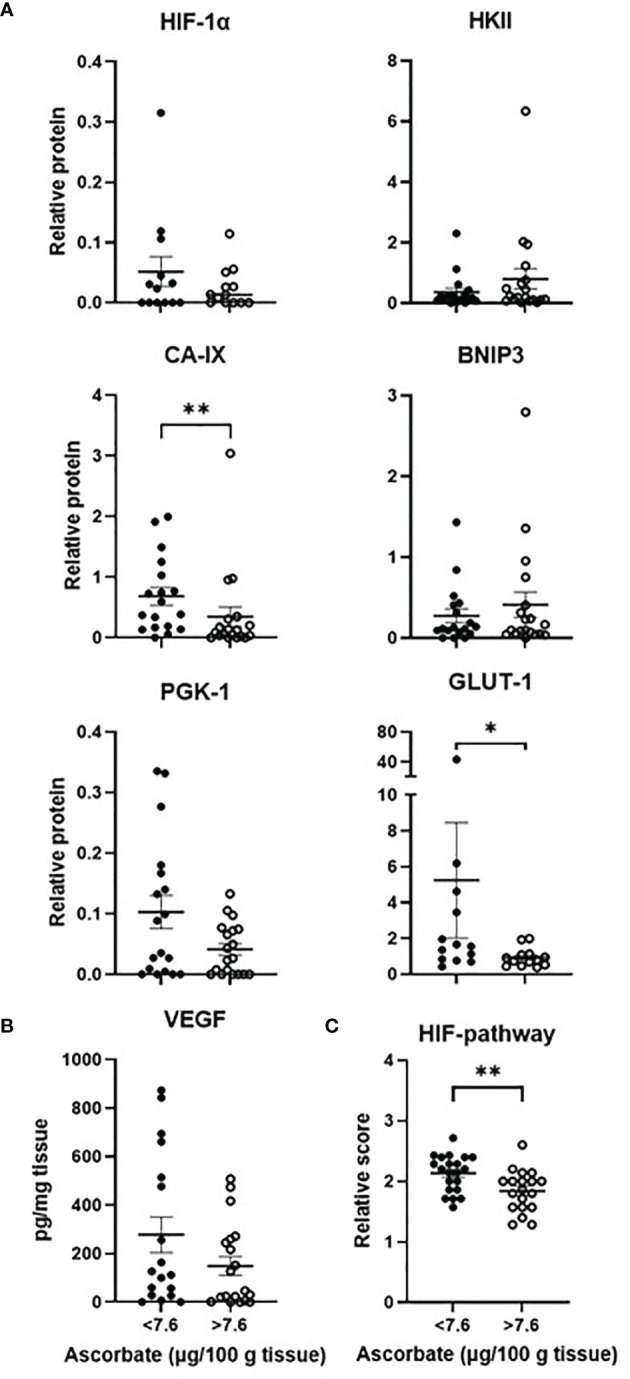
The hypoxic pathway according to ascorbate content. The cohort was divided into tumours with below or above median ascorbate (7.6 μg/100 mg tissue), showing members of the HIF-pathway **(A)** estimated by Western blotting or **(B)** ELISA. Protein levels were not normally distributed (Shapiro-Wilk test), hence Mann Whitney test was used to calculate significance. Levels of all 7 proteins were divided in to low, medium or high expression to derive a relative HIF-pathway score for each tumour. Tumours with above median ascorbate had significantly lower HIF-pathway score **(C)**. The relative HIF-pathway scores were normally distributed (Shapiro-Wilk test), hence unpaired t test was used to calculate significance. n=37 samples; mean ± SEM; *p < 0.05, **p < 0.01.

The cohort of samples was divided into two groups according to median tissue ascorbate levels (7.6 μg ascorbate/100 mg tissue, **Figures 3A, B** **)**. CA-IX (Mann-Whitney, p = 0.006) and GLUT1 (Mann-Whitney, p = 0.030) were significantly lower in samples with above median tissue ascorbate. Although HIF-1α, PGK1 and VEGF tended to be lower in tumours with above median ascorbate, significance was not reached. Tumours with above median ascorbate levels had a significantly lower HIF pathway score than those with below median ascorbate levels (unpaired t test, p = 0.007, **Figure 3C**). Results were similar when ascorbate was standardised to DNA, with the association with both CA-IX and VEGF reaching significance (Mann-Whitney, 0 = 0.018 and p = 0.009, respectively, **Supplementary Figure 4**).

Spearman’s correlation was used to assess the relationship between ascorbate and hypoxic pathway protein levels. CA-IX (Spearman r -0.45, p = 0.005) and GLUT1 (Spearman -0.55, p = 0.004) were negatively correlated with tissue ascorbate (**Table 3**), and BNIP3 and VEGF tended to be negatively correlated with cellular ascorbate (both Spearman r -0.31, p = 0.06) (**Table 3**). There was a negative correlation between tissue ascorbate and the HIF pathway score (Pearson r -0.327, p = 0.048).

### Patient Survival

Treatment and follow-up data were obtained from patients’ clinical notes. For patients lost to follow-up (2/37), the time from surgical resection (and tissue donation) until the patient was last seen alive was used for generating survival data. Of our patients, 33/37 were confirmed to have died, whereas 4/37 were either still alive or were lost to follow-up. Furthermore, 15/37 had recorded progression/recurrence, with no progression information for the remainder. Survival analysis was therefore done for overall survival, assumed to be disease-specific survival, and not for progression-free survival.

Standard post-surgery therapy included 60 Gy radiation in 30 fractions and temozolomide chemotherapy in 6 cycles (**Table 1**). Patients with glioblastoma had a median overall survival of 335 days (~ 11 months). Median survival for patients treated with radiation alone was 225.5 days (n=12), with chemoradiation was 407 days (n=18), and for those without any post-surgery treatment 128 days (n = 4). As expected, univariate analysis demonstrated that radiation, chemotherapy (temozolomide) or any therapy post-surgery (mostly concurrent chemoradiation) were all associated with significantly better survival (HR 0.107, 0.218 and 0.078, respectively, **Supplementary Table 1**).

Interestingly, according to patient medical notes, three patients had independently sought high dose vitamin C infusions after surgery, but no details of dose or duration were available. As infusions were carried out after sample collection, ascorbate content post infusion was not available. Impact on survival of ascorbate infusions could not be statistically assessed; for these three individuals, the days from surgery to death were 335 and 567, and days from surgery to last seen alive were 347. All three patients had also received standard chemoradiation.

**Figure 4:**
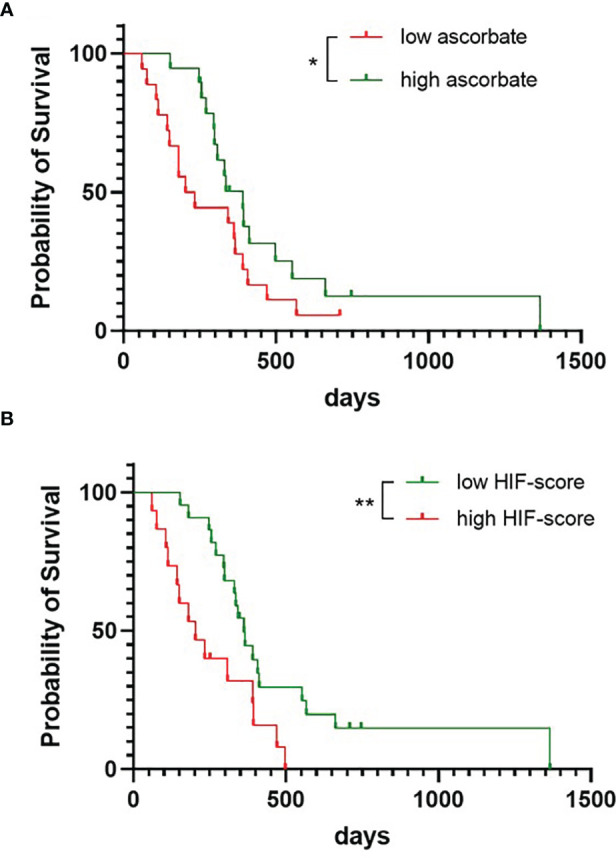
Survival probability of patients with glioblastoma. The cohort was divided into patients with tumours with below or above median ascorbate (7.6 μg/100 mg tissue) **(A)**, or with tumours with below or above median HIF-pathway score **(B)**, presented as Kaplan-Meier curves, and analysed using Gehan-Breslow-Wilcoxon test. n = 37 patients; *p < 0.05, **p < 0.01.

Survival according to tumour ascorbate levels was assessed by separating the cohort into patients with above or below median tissue ascorbate levels (7.6 μg ascorbate/100 mg tissue). Survival for patients with above median tissue ascorbate levels was significantly longer than for patients with below median tissue ascorbate levels (Gehan-Breslow-Wilcoxon p = 0.027, **Figure 4A**). Median survival for the above median ascorbate group was 390 days, compared to the below median ascorbate group with 219 days. Univariate Cox regression analysis similarly indicated a survival advantage for patients with above median tumour ascorbate, although significance was lost (**Supplementary Table 1**, HR 0.555, p = 0.098). Age, gender and IDH1 status were not significantly associated with survival (**Supplementary Table 1**).

We noticed that ascorbate appeared to play more of a role early in the follow-up period. Univariate analysis confirmed a shift from HR 0.093, p = 0.025 at 180 days, to HR 0.133, p = 0.009 at 250 days, to HR 0.467, p = 0.081 at 365 days and HR 0.555, p = 0.098 at 730 days (**Supplementary Table 1**). We have analysed the first 250 days further, using multivariate regression analyses, which showed that the ascorbate-related survival advantage (HR 0.133, p = 0.009) remained even after adjusting for age (HR 0.167, p = 0.023) (**Supplementary Table 2**).

The below median ascorbate group contained 4 patients who did not receive any therapy and 6 who only received radiation and no chemotherapy (and one without treatment information), whereas the above median ascorbate group contained patients who all received radiation, and 5 also received chemotherapy (and one without treatment information). Accordingly, multivariate analysis (censored at 250 days) showed that survival advantage according to ascorbate was lost when adjusted for treatment (HR 0.214, p = 0.06) or for age with treatment (HR 0.244, p = 0.087) (**Supplementary Table 2**).

Survival for patients with below median HIF-pathway score tumours was significantly longer than for patients with above median scores (Gehan-Breslow-Wilcoxon p = 0.005, **Figure 4B**). Median survival for the low HIF-pathway score group was 362 days compared to 203 days for the high HIF-pathway score group. Univariate analysis showed that a low HIF-pathway score was significantly associated with improved survival for most of the follow-up period (HR 0.144, p = 0.016 at 180 days, HR 0.152, p = 0.005 at 250 days, HR0.463, p = 0.075 at 365 days and HR 0.392, p = 0.013 at 730 days, **Supplementary Table 1**). Multivariate analysis (for follow-up censored at 250 days) showed that the HIF-pathway score remained significant even after adjusting for age (HR 0.139, p = 0.004) or for age with treatment (HR 0.207, p = 0.035), although significance was lost when adjusted for treatment alone (HR 0.255, p = 0.062) (**Supplementary Table 2**).

## Discussion

In this study we report the concentration of ascorbate in clinical glioblastoma samples, show a strong association between ascorbate and HIF-pathway activity, and describe improved survival for patients with glioblastomas that contain higher levels of ascorbate or lower HIF-pathway activity.

Although it is known that the brain contains among the highest concentration of ascorbate in the human body (~14 μg/100 mg tissue, 57), this is the first study to measure the vitamin in glioblastoma. One previous study in eleven patients reported ascorbate levels in lower-grade astrocytomas (9.4 ± 2.3 μg/100 mg tissue or 0.309 ± 0.068 nmol/μg DNA) and adjacent non-neoplastic brain (9.4 ± 1.5 μg/100 mg or 0.552 ± 0.092 nmol/μg DNA) (58). Our findings in glioblastoma (7.61 ± 0.84 μg/100 mg or 0.446 ± 0.063 nmol/μg DNA) are comparable to the astrocytoma levels, and potentially lower than those in non-neoplastic tissue. Due to ethical consideration, uninvolved tissue is not collected during surgical resection for glioblastoma and was not available for the current investigation.

Ascorbate enters the brain primarily *via* transport from plasma across the epithelium of the choroid plexus to the cerebrospinal fluid, with cellular uptake from this fluid occurring *via* the sodium vitamin C cotransporters (SVCTs) (56). It was of interest to see in our study that glioblastomas arising in different parts of the brain appeared to contain different ascorbate levels, with those located in the parietal lobes containing the lowest ascorbate levels, and those located in the temporal lobes, the highest ascorbate levels. Limited information is available on the distribution of ascorbate in the brain, but analysis of guinea pig brain tissue indicated lower levels in the hippocampus compared with the frontal cortex and cerebellum (60). Ascorbate levels in the pituitary are much higher than in brain (40-50 mg/100 g tissue) (61). A previous study reported that patients with temporal lobe gliomas (both low- and high-grade gliomas) had poorer survival than those with gliomas at other locations (62). Other studies found that gliomas with IDH mutations tended to be located within the frontal or temporal lobes, but rarely in the diencephalon or brain stem (63, 64). In our study, two IDH1 mutant gliomas were located in the frontal, and one in the temporal lobe, all three with above median ascorbate content.

The predominant IDH mutation, IDH1^R132H^, is common in low-grade gliomas (>70%) and ‘secondary’ glioblastomas (>80%, astrocytoma grade 4), but rare in ‘primary’ glioblastoma (12%) (2, 4). Mutant IDH1 enzymes exhibit gain-of-function activity, converting isocitrate to D-2-hydroxyglutarate (D-2-HG), which inhibits activity of 2-OGDDs (65, 66). As our glioblastoma cohort contained only 3 IDH1 mutant tumours, the impact of the IDH1^R132H^ mutation on HIF-hydroxylase activity, or on the association between ascorbate and the HIF-pathway, is intriguing but could not be assessed.

We observed a strong inverse relationship between ascorbate content and HIF-pathway activity, similar to reports in other cancer types (49–53), providing further evidence that ascorbate plays an important role in regulating the HIF-hydroxylases in cancer. In our study with glioblastoma, this relationship was largely driven by CA-IX, GLUT-1 and VEGF, which have all previously been linked to higher grade of glioma and poorer patient survival (67, 68). Protein levels of HIF-1α correlated positively with BNIP3 but poorly with most other hypoxia regulated proteins. BNIP3 also correlated with HKII, and VEGF with both CA-IX and PGK1, as expected. The inverse relationship between BNIP3 and PGK1 was unexpected, and together with the minimal associations seen between several other proteins, suggests a multi-level regulatory system of the hypoxic pathway in glioblastoma.

HIF-1 and its isoform HIF-2 bind to identical recognition sequences in the genome, but their response to hypoxia, their tissue distribution and target genes are different (69). There is limited information on HIF-2α in glioblastoma, but recent clinical trials using specific HIF-2α inhibitors (such as MK-6482 or PT-2385) in other cancer types has sparked clinical interest (70, 71). However, HIF-2α protein was not detected in any of our samples and was therefore not pursued further.

The original study on the effect of concomitant radiation with temozolomide (7) reported median overall survival of 12.1 months for radiotherapy and 14.6 months for chemoradiation, compared to survival of our cohort (overall survival of 11 months, radiotherapy 7.5 months, chemoradiation 13.6 months). The original study contained only patients fit enough to be entered on the trial while some of ours could have had poorer fitness, some due to more advanced tumours, especially those receiving radiation alone.

Lower HIF-pathway activity was associated with longer patient survival, agreeing with previous glioblastoma studies (23, 40). This survival advantage remained strong even after adjustment for age and for age with treatment, suggesting a robust association. We also saw an association between above median ascorbate levels and longer patient survival, which agrees with our previous findings in colorectal and breast cancer (50, 53). The survival advantage according to higher tumour ascorbate levels appeared to be more prominent for the first 8 months following surgery, but the reasons for this are unknown. Together with the strong inverse association of ascorbate and the HIF pathway, the use of ascorbate as a potential therapeutic intervention in hypoxic glioblastomas seems promising. However, this study does not provide evidence for a causative relationship, and therefore further research is required before use of ascorbate as a treatment option can be recommended.

We were unable to determine whether tumour ascorbate levels were governed by ascorbate supply (via reduced dietary intake), or by ascorbate uptake (via SVCTs), as only tumour tissue was available for analysis, and no (appropriately processed (59)) plasma. Cellular ascorbate uptake occurs primarily *via* SVCT2 (56), but SVCT2 protein levels or *SLC23A2* expression have not been reported for glioblastoma. In addition, SVCT1/2 protein levels do not necessarily predict ascorbate accumulation in tumour tissue (72). GLUT1 can take up the oxidised form, dehydroascorbate [DHA (73)], in competition with glucose, but as *in vivo* levels of DHA are <10% of total ascorbate (59, 74), this mechanism is unlikely to play an important role. In addition, in our study, ascorbate content was inversely correlated with GLUT1 protein levels.

Three of the patients in our study had reported accessing high dose ascorbate infusions after resection (and tissue donation), but post-infusion tumour ascorbate content, and any impact on survival, are unknown. Future prospective studies are required to determine why some glioblastomas accumulate more ascorbate than others, whether ascorbate uptake declines during tumour progression, and whether increasing ascorbate supply, *via* dietary means or high dose ascorbate infusions, could increase glioblastoma ascorbate content, as we have shown in colorectal tumours (54).

Ascorbate is a potent antioxidant, and this activity of reducing reactive oxygen species may play an important role in cancer progression and response to treatment (75, 76). Ascorbate is also an essential cofactor for the 2-OGDD enzymes that encompass not only the HIF-hydroxylases, but also the ten-eleven translocases (TETs) that demethylate DNA (reviewed in 77). The ability of ascorbate to increase TET function has been investigated in a number of preclinical studies, most commonly for acute myeloid leukaemia, but also in renal cell carcinoma. In these studies, reduced ascorbate was associated with reduced TET function, leading to leukemogenesis (78–80). In gliomas, the TETs may play a particularly important role as DNA hypermethylation is a good prognostic indicator (81), and suppression of DNA repair *via* methylation of the O-6-methylguanine-DNA methyltransferase (MGMT) promoter predicts response to temozolomide (82). DNA methylation with respect to ascorbate content of glioma samples will be addressed in a separate manuscript.

Our study has a number of limitations to consider, including relatively low sample numbers and analysis of resectable tumours only, which excludes tumours too deeply embedded or near critical brain structures. In addition, patients with higher tumour ascorbate levels tended to receive more comprehensive therapy of both radiation and temozolomide in this retrospective study. Therefore, higher tumour ascorbate content may instead predict those patients who are more able to receive full treatment, whereas low tumour ascorbate content may indicate rapid patient deterioration. The high ascorbate group also contained all three IDH1 mutant tumours, which are known to have a better prognosis (4). However, Cox regression analysis showed that IDH1 mutation status did not significantly impact survival in this cohort of glioblastoma.

## Conclusion

This retrospective observational study provides compelling evidence of an inverse relationship between ascorbate content and hypoxic pathway activity in glioblastoma, and that survival is poorer when tumours have an active hypoxic pathway. We provide data showing an association between improved survival and higher tumour ascorbate content, but only intervention trials with suitable controls will be able to assess whether increasing ascorbate in glioblastoma can affect patient survival.

## Data Availability Statement

The raw data supporting the conclusions of this article will be made available by the authors, without undue reservation.

## Ethics Statement

The studies involving human participants were reviewed and approved by University of Otago Ethics Committee (H19/163) and the national Health and Disability Ethics Committee (MEC/08/02/016). The patients/participants provided their written informed consent to participate in this study. The animal study was reviewed and approved by the University of Otago Animal Ethics Committee.

## Author Contributions

EB processed and analysed samples. RC and GW analysed DNA and IDH status. HM curated sample collection and retrieved patient clinical data. JR and TS provided additional samples and advice on gliomas. MV provided oversight of ascorbate biochemistry and BR provided clinical oversight. EB, EP and GD analysed data. GD, EP and BR attracted funding. GD designed and coordinated the study. EB and GD wrote the draft manuscript. All authors contributed to editing the manuscript and approved the submitted version.

## Funding

Funding was obtained from the Canterbury Medical Research Foundation, the University of Otago Research Grant and the Mackenzie Charitable Foundation.

## Conflict of Interest

The authors declare that the research was conducted in the absence of any commercial or financial relationships that could be construed as a potential conflict of interest.

## Publisher’s Note

All claims expressed in this article are solely those of the authors and do not necessarily represent those of their affiliated organizations, or those of the publisher, the editors and the reviewers. Any product that may be evaluated in this article, or claim that may be made by its manufacturer, is not guaranteed or endorsed by the publisher.

## Abbreviations

2-OGDD, 2-oxoglutarate dependent dioxygenase; BNIP3, BCL2/adenovirus E1B 19 kDa protein-interacting protein 3; CA-IX, carbonic anhydrase-9; CSTB, Cancer Society Tissue Bank; DHA, dehydroascorbate; FIH, factor inhibiting HIF; GAPDH, glyceraldehyde 3-phosphate dehydrogenase; GLUT1, glucose transporter 1; HIF, hypoxia inducible factor; HKII, hexokinase 2; HPLC-ECD, high performance liquid chromatography with electrochemical detection; IDH, isocitrate dehydrogenase; PGK1, phosphoglycerate kinase 1; PHD, prolyl hydroxylase; SVCT, sodium vitamin C cotransporter; VEGF, vascular endothelial growth factor; WHO, World Health Organisation.
