# Supplementary material for: Increased Ascorbate Content of Glioblastoma Is Associated With a Suppressed Hypoxic Response and Improved Patient Survival
Source: Front Oncol. 2022 Mar 28;12:829524. doi: 10.3389/fonc.2022.829524 (PMC8995498; doi:10.3389/fonc.2022.829524)
Supplement: Supplementary file 1 [file DataSheet_1.pdf]

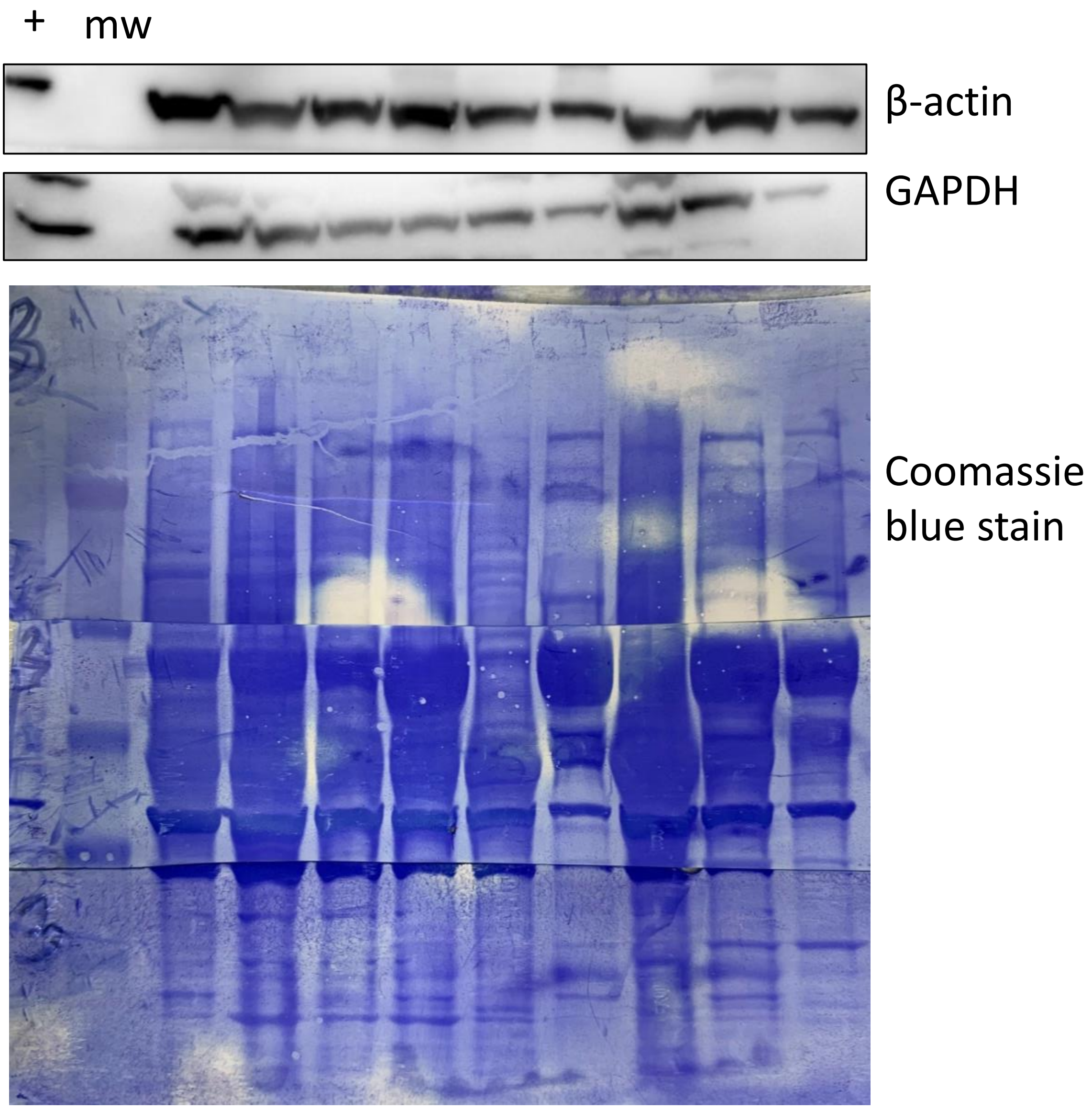

Supplementary Figure 1. Western blot images showing an example of staining for β-actin, GAPDH and total protein from the same blot. The blot contained samples from 9 individual tumours, as well as a positive control (MDA-MB-231 cell line treated with 50 μM cobalt chloride) and molecular weight (mw).

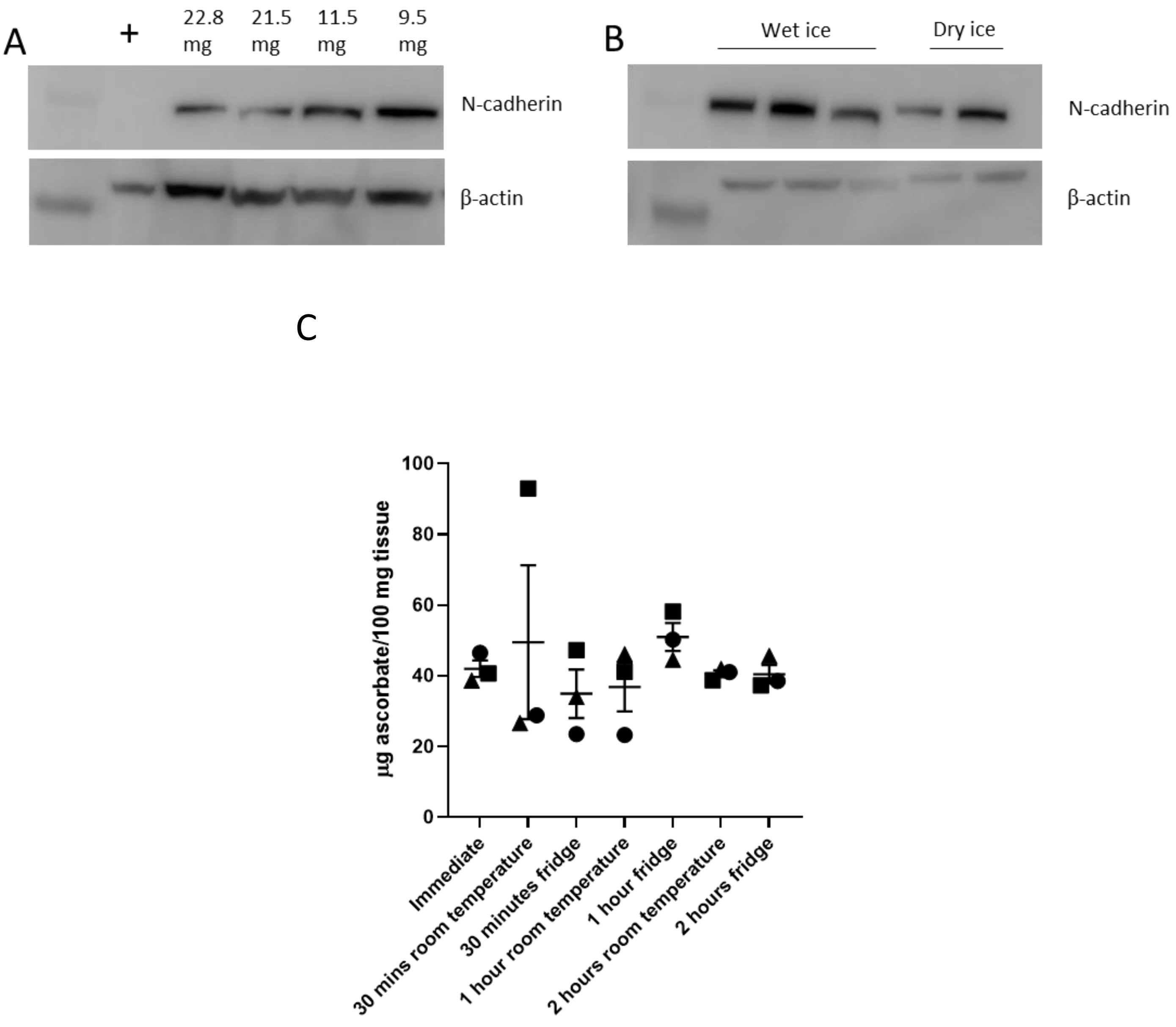

Supplementary Figure 2. Optimisation of techniques using mouse brain. Required samples weight to be processed was shown to be around 10 mg (A). Samples processed on wet ice and dry ice both showed sufficient protein content for analysis (B), and processing of samples within 2 hours did not reduce ascorbate content, although variability was noticeable (C). Western blots were stained for N-cadherin and β-actin, and samples were analysed for ascorbate using HPLC-ECD. Three mouse brains were processed for each time point, mean ± SEM.

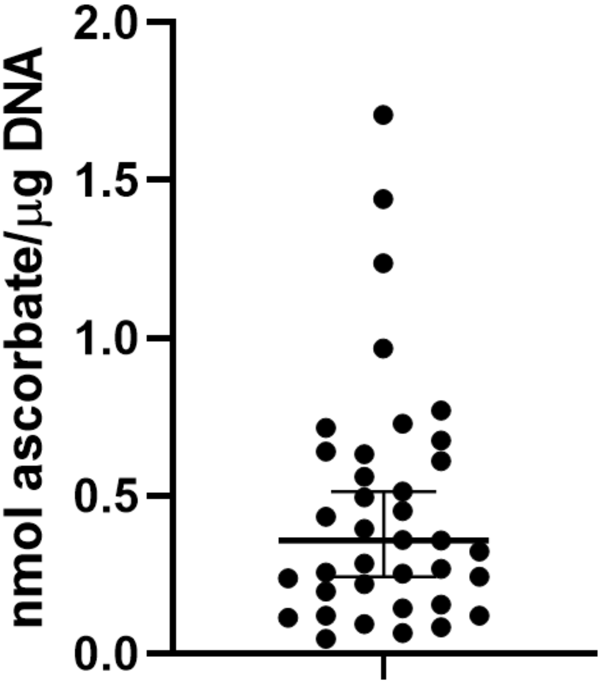

Supplementary Figure 3. Ascorbate content of glioblastoma tumours. Ascorbate was measured by HPLC-ECD and standardised to DNA (cellular ascorbate). N=37 samples

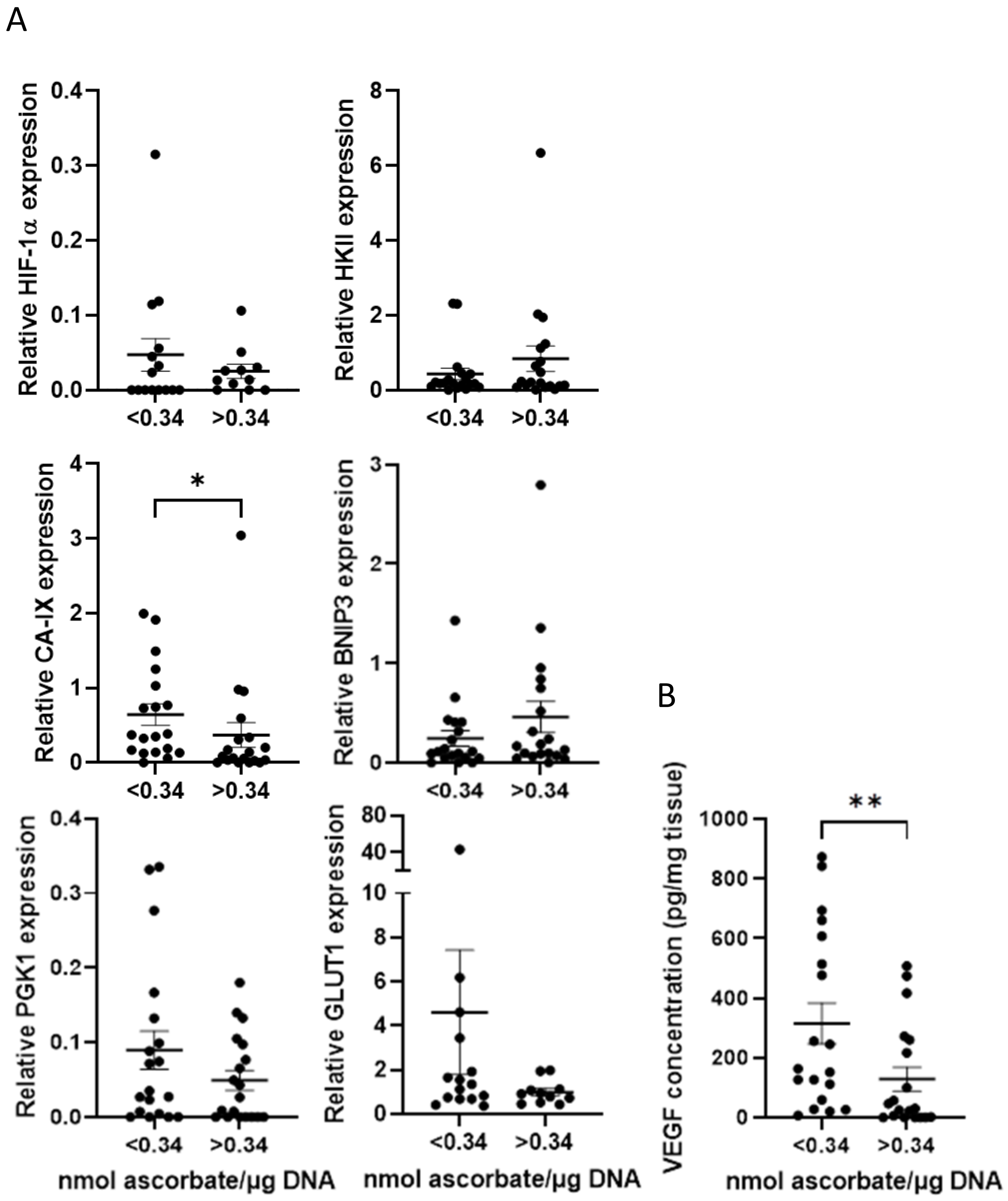

Supplementary Figure 4. The hypoxic pathway according to ascorbate content. The cohort was divided into tumours with below or above median ascorbate (0.34 nmol/µg DNA), showing members of the HIF-pathway (A) estimated by Western blotting or (B) ELISA. n=37 samples; mean ± SEM; * $p < 0.05$, ** $p < 0.01$.
