# Supplementary material for: Increased Ascorbate Content of Glioblastoma Is Associated With a Suppressed Hypoxic Response and Improved Patient Survival
Source: Front Oncol. 2022 Mar 28;12:829524. doi: 10.3389/fonc.2022.829524 (PMC8995498; doi:10.3389/fonc.2022.829524)
Supplement: Supplementary file 2 [file DataSheet_2.pdf]

Supplementary Table 1: Univariate regression analysis of survival of patients with glioblastoma censored at different times of follow-up.

	Censored at 180 days				Censored at 250 days				Censored at 365 days				Censored at 730 days			
**Variable**	**HR**	**lower**	**upper**	**p**	**HR**	**lower**	**upper**	**p**	**HR**	**lower**	**upper**	**p**	**HR**	**lower**	**upper**	**p**
**↑Asc**	0.093	0.012	0.743	**0.025**	0.133	0.029	0.610	**0.009**	0.467	0.199	1.098	0.081	0.555	0.276	1.116	0.098
**↓HIF**	0.144	0.030	0.697	**0.016**	0.152	0.041	0.566	**0.005**	0.463	0.198	1.080	0.075	0.392	0.188	0.818	**0.013**
**Age**	1.071	0.999	1.147	0.053	1.069	1.009	1.134	**0.025**	1.037	0.997	1.079	0.070	1.010	0.978	1.043	0.535
**Sex**	0.479	0.129	1.785	0.273	0.362	0.117	1.125	0.079	0.606	0.254	1.448	0.260	0.537	0.254	1.136	0.104
**IDH1**	1.3E-08	0	Inf	0.998	1.3E-08	0	Inf	0.998	0.469	0.063	3.489	0.459	0.660	0.157	2.779	0.571
**Rad**	0.131	0.034	0.501	**0.003**	0.107	0.031	0.363	**0.0003**	0.107	0.031	0.363	**0.0003**	0.107	0.031	0.363	**0.0003**
**Chemo**	3.3E-10	0	Inf	0.999	0.046	0.006	0.361	**0.003**	0.214	0.087	0.532	**0.001**	0.218	0.097	0.490	**0.0002**
**Therapy**	0.078	0.020	0.309	**0.0003**	0.078	0.020	0.309	**0.0003**	0.078	0.020	0.309	**0.0003**	0.078	0.020	0.309	**0.0003**

Asc, ascorbate; HIF, hypoxia inducible factor 1 pathway; Rad, radiation therapy; chemo, temozolomide chemotherapy; Therapy, all: radiation, chemotherapy and chemoradiation; HR, hazard ratio; lower and upper, 95% confidence intervals from Cox regression analysis.

Supplementary Table 2: Univariate and multivariate analysis of glioblastoma survival censored at 250 days.

	Univariate				Adjusted for Age				Adjusted for Treatment				Adjusted for Age + Treatment			
**Variable**	**HR**	**lower**	**upper**	**p**	**HR**	**lower**	**upper**	**p**	**HR**	**lower**	**upper**	**p**	**HR**	**lower**	**upper**	**p**
**↑Asc**	0.133	0.029	0.610	**0.009**	0.167	0.036	0.780	**0.023**	0.214	0.043	1.066	0.060	0.244	0.049	1.229	0.087
**↓HIF**	0.152	0.041	0.566	**0.005**	0.139	0.036	0.532	**0.004**	0.255	0.061	1.068	0.062	0.207	0.048	0.893	**0.035**
**Age**	1.069	1.009	1.134	**0.025**					1.054	0.987	1.127	0.117				
**Sex**	0.362	0.117	1.125	0.079	0.310	0.096	0.996	**0.049**	0.509	0.156	1.659	0.262	0.430	0.127	1.456	0.175
**IDH1**	1.3E-08	0	Inf	0.998	4.5E-08	0	Inf	0.998	7.8E-09	0	Inf	0.999	2.0E-08	0	Inf	0.999
**Rad**	0.107	0.031	0.363	**0.000**	0.114	0.032	0.403	**0.001**								
**Chemo**	0.046	0.006	0.361	**0.003**	0.053	0.007	0.423	**0.006**								
**Therapy**	0.078	0.020	0.309	**0.000**	0.107	0.026	0.438	**0.002**								

Asc, ascorbate; HIF, hypoxia inducible factor 1 pathway; Rad, radiation therapy; chemo, temozolomide chemotherapy; Therapy, all: radiation, chemotherapy and chemoradiation; HR, hazard ratio; lower and upper, 95% confidence intervals from Cox regression analysis.
